# Anterograde and Retrograde Propagation of Inoculated Human Tau Fibrils and Tau Oligomers in a Non-Transgenic Rat Tauopathy Model

**DOI:** 10.3390/biomedicines11041004

**Published:** 2023-03-24

**Authors:** Lea Langer Horvat, Ena Španić Popovački, Mirjana Babić Leko, Klara Zubčić, Luka Horvat, Maja Mustapić, Patrick R. Hof, Goran Šimić

**Affiliations:** 1 Department of Neuroscience, Croatian Institute for Brain Research, University of Zagreb School of Medicine, 10000 Zagreb, Croatia; 2 Department of Molecular Biology, Faculty of Science, University of Zagreb, 10000 Zagreb, Croatia; 3 Laboratory of Clinical Investigation, National Institute on Aging Intramural Research Program, National Institutes of Health, Baltimore, MD 21224, USA; 4 Nash Family Department of Neuroscience, Friedman Brain Institute, and Ronald M. Loeb Center for Alzheimer’s Disease, Icahn School of Medicine at Mount Sinai, New York, NY 10029, USA

**Keywords:** Alzheimer’s disease, cognition, neurofibrillary degeneration, spatial working memory, tau fibrils, tau oligomers, tau protein, tauopathy model, Wistar rat

## Abstract

The tauopathy of Alzheimer’s disease (AD) is first observed in the brainstem and entorhinal cortex, spreading trans-synaptically along specific pathways to other brain regions with recognizable patterns. Tau propagation occurs retrogradely and anterogradely (trans-synaptically) along a given pathway and through exosomes and microglial cells. Some aspects of in vivo tau spreading have been replicated in transgenic mice models expressing a mutated human *MAPT* (tau) gene and in wild-type mice. In this study, we aimed to characterize the propagation of different forms of tau species in non-transgenic 3–4 months old wild-type rats after a single unilateral injection of human tau oligomers and tau fibrils into the medial entorhinal cortex (mEC). We determined whether different variants of the inoculated human tau protein, tau fibrils, and tau oligomers, would induce similar neurofibrillary changes and propagate in an AD-related pattern, and how tau-related pathological changes would correlate with presumed cognitive impairment. We injected human tau fibrils and tau oligomers stereotaxically into the mEC and examined the distribution of tau-related changes at 3 days and 4, 8, and 11 months post-injection using antibodies AT8 and MC1, which reveal early phosphorylation and aberrant conformation of tau, respectively, HT7, anti-synaptophysin, and the Gallyas silver staining method. Human tau oligomers and tau fibrils exhibited some similarities and some differences in their ability to seed and propagate tau-related changes. Both human tau fibrils and tau oligomers rapidly propagated from the mEC anterogradely into the hippocampus and various parts of the neocortex. However, using a human tau-specific HT7 antibody, 3 days post-injection we found inoculated human tau oligomers in the red nucleus, primary motor, and primary somatosensory cortex, a finding not seen in animals inoculated with human tau fibrils. In animals inoculated with human tau fibrils, 3 days post-injection the HT7 antibody showed fibrils in the pontine reticular nucleus, a finding explained only by uptake of human tau fibrils by incoming presynaptic fibers to the mEC and retrograde transport of inoculated human tau fibrils to the brainstem. Rats inoculated with human tau fibrils showed as early as 4 months after inoculation a spread of phosphorylated tau protein at the AT8 epitopes throughout the brain, dramatically faster propagation of neurofibrillary changes than with human tau oligomers. The overall severity of tau protein changes 4, 8, and 11 months after inoculation of human tau oligomers and tau fibrils correlated well with spatial working memory and cognition impairments, as measured by the T-maze spontaneous alternation, novel object recognition, and object location tests. We concluded that this non-trangenic rat model of tauopathy, especially when using human tau fibrils, demonstrates rapidly developing pathologic alterations in neurons, synapses, and identifiable pathways together with cognitive and behavioral changes, through the anterograde and retrograde spreading of neurofibrillary degeneration. Therefore, it represents a promising model for future experimental studies of primary and secondary tauopathies, especially AD.

## 1. Introduction

Tau protein is mainly expressed in neurons where it plays an important role in assembling and stabilizing the microtubules [1]. Phosphorylation regulates the physiological function of tau by controlling its binding to microtubules [2]. Under pathological conditions, tau becomes abnormally phosphorylated, and its accumulation leads to its misfolding and oligomerization into insoluble neurofibrillary tangles (NFT) as well as neuropil threads, which are the pathological hallmarks of tauopathies [3,4,5]. Tau protein exists in six isoforms ranging from 352 to 441 amino acids in length that are generated by alternative mRNA splicing from a single *MAPT* (tau) gene and are expressed in the adult human brain as well as in the rat brain [6,7].

The spatiotemporal pattern of tau pathology progression indicates that the progression or spreading of tau pathology is likely mediated trans-synaptically by transmission from one region of the brain to other anatomically connected regions [8,9]. Increasing in vitro and in vivo evidence shows that templated propagation of tau pathology includes active neuron-to-neuron transfer, seeding-misfolding induction of molecular conformation of the endogenous protein into a pathological form, and propagation-transport of the misfolded protein through neuroanatomically linked brain regions [10]. However, the species of tau needed to promote aggregation or seeding remains unclear. Different tau phosphorylation patterns, isoforms, species, and aggregates are identified among tauopathies and different tau strains might transfer tau pathology from cell to cell [11,12]. Although Mirbaha and coworkers identified a stable form of tau monomer that is seed-competent, most studies showed that soluble oligomeric tau is the most seeding-prone species but preparation and definition of human tau oligomers vary considerably among different groups [13,14,15,16]. For example, Kayed and colleagues generated human tau oligomers by cross-seeding with amyloid β (Aβ) oligomers, and those oligomers induced cell death when added exogenously to SH-SY5Y cells [17,18].

With the recent definition of Alzheimer’s disease (AD) as a biological spectrum, using the National Institute on Aging/Alzheimer’s Association (NIA-AA) research framework [19,20], it is now possible to classify different pathophysiologically defined subtypes of AD [21]. A clinicopathological study distinguished three AD subtypes based on postmortem NFT density: typical AD with balanced NFT counts in the neocortex and hippocampus (75%), hippocampal-sparing forms with predominant involvement of association cortices (11%), and a limbic-predominant type with predominant involvement of the hippocampus (14%) [22]. AD subgroups, based on the distribution of tau pathology and corresponding brain atrophy have also been identified by many neuroimaging studies using structural magnetic resonance imaging (MRI) and tau-positron emission tomography (PET) [23,24,25,26]. The pathogenic factors underlying AD subtypes are nevertheless still unclear and cannot be explained by pathological changes of Aβ peptide alone because the distribution of Aβ PET retention is quite similar in all subtypes [27]. There is increasing evidence that Aβ can assemble into distinct strains of aggregates, which may be the primary driver of the phenotypic heterogeneity of AD. This indicates that AD subtypes may be linked to different tau protein modifications, suggesting that these patients may have multiple molecular drivers of an otherwise common phenotype. A recent study using tau PET scans from 1612 individuals identified four distinct spatiotemporal trajectories of tau pathology [28]. It replicated the previously described limbic-predominant and hippocampal-sparing patterns and discovered posterior cortical atrophy and lateral-temporal patterns resembling atypical clinical variants. These subtypes present with distinct demographic and cognitive profiles and differing longitudinal outcomes. In addition, network diffusion models implied that pathology originates and spreads through distinct corticolimbic networks in the different subtypes, suggesting that variation in tau pathology is common and systematic [25,26]. Although the Braak and Braak staging system and NIA-AA criteria do not include their evaluation, several studies drew attention to the possibility of selective and early involvement of the brainstem nuclei, particularly locus coeruleus and raphe nuclei, in the pathogenesis of AD [29,30,31]. Accumulation of intracellular tau protein aggregates and neurofibrillary lesions strongly correlate with cognitive decline in AD patients and are considered to be a central pathogenetic mechanism of AD [32,33,34,35]. Similar slower or faster accumulation and spread of pathologically modified tau through connected neuronal circuits has been identified in other neurodegenerative tauopathies, such as argyrophilic grain disease progressive supranuclear palsy, and frontotemporal dementia [36,37,38].

De Calignon et al. and Liu et al. used mouse models in which expression of human tau was restricted only to the entorhinal cortex (EC), but tau pathology nevertheless propagated to brain regions anatomically linked to the EC [8]. Other authors have also reported the appearance of tau pathology in areas connected to the sites injected with tau seeds or viral vectors expressing tau [39,40,41,42]. These studies suggest that tau transmission occurs over long distances, along axons that are efferent and afferent to the injection site, rather than as a result of the proximity of neurons to the injection site [8,9,39,43].

Templated propagation of tau pathology has been investigated in many in vitro and in vivo models [44,45,46,47]. Several studies have assessed the transmissibility and seeding of tau pathology using a variety of different approaches in transgenic mice, mainly by injection of brain extracts from animal models, AD brains, or other tauopathies [12,47,48]. Briefly, Clavaguera and coworkers showed that injecting tau aggregates extracted from mice overexpressing mutated tau (P301S) into mice overexpressing human wild-type tau was sufficient to induce tau pathology in anatomically connected brain regions, and reproduced the morphology of the lesions seen in human disease in rodent brains by injecting human brain homogenates of different tauopathies [12,47]. Injections of artificial or brain-derived seeds in transgenic animals potentiate transmissibility [39,41,49,50,51]. In these mouse models, human-mutated tau seeds are overexpressed, and additional seeds are injected. However, the injected seeds require additional transgenic overexpression of murine tau, either wild-type or altered, to drive disease progression [12,52].

Current transgenic mice tauopathy models predominantly express tau protein with a mutation at P301S or P301L, observed exclusively in frontotemporal dementia with parkinsonism linked to chromosome 17 (FTDP-17) in which neurons undergo rapid neurofibrillary degeneration over only a few weeks [42,43,47,53]. However, in most tauopathies neurofibrillary degeneration is a very slow process [54], suggesting a substantial difference in the underlying mechanisms of aggregation. Therefore, transgenic models expressing mutated or truncated tau have a time course of tau aggregation and neuronal death not comparable to classic sporadic tauopathies. It is therefore logical to conclude that there are functional differences between the wild-type and mutated forms of tau that cause differences in the molecular mechanisms between these models and human tauopathies [55,56]. Also, intracerebral inoculation of a high amount of tau fibrils purified from AD brains, but not synthetic tau fibrils, resulted in the formation of tau pathology in anatomically connected brain regions in non-transgenic mice [57]. In a later study, the injection of tau protein induced tau pathology, as well as its spreading in non-transgenic mice, suggesting that higher levels of pathological tau injected into the brain of wild-type mice are essential for the induction and spread of tau pathology [58,59]. Most recently, it has been hypothesized that tau pathology within selected projection neurons with susceptible microenvironments can initiate sporadic AD, where abnormally phosphorylated tau on microtubules traps APP-containing endosomes, which increases Aβ production and drive a vicious cycle over a lifespan [60].

In this study, we analyzed the propagation of different forms of tau species in a non-transgenic wild-type rat to determine whether tau progression is similar to an AD-related pattern, and to assess the possible association of tau pathology with cognitive impairment. As the EC is the brain area affected earliest and most severely by tau pathology in most AD cases [61,62], and to develop a non-transgenic rat model of sporadic AD, we injected human tau oligomers and tau fibrils into the medial entorhinal cortex (mEC).

## 2. Materials and Methods

### 2.1. Animals

Three to four months old male Wistar rats were used. The animals were purchased from the Institute for Medical Research and Occupational Health, Zagreb, Croatia. All animals were housed in a temperature-controlled room and maintained on a 12-day/night cycle with food and water provided ad libitum. All experiments were performed with the approval of the Ethical Committee of the University of Zagreb Faculty of Science (EP 02/2015 from 15 August 2015) and by relevant laws (Animal Welfare Law 135/06 and 37/13) and regulations of the Ministry of Agriculture of the Republic of Croatia (approval no. NP-999/15-01/15 from 12 October 2015) and EU guidelines for the use of animals for scientific purposes formulated in the European Directive 2010/63/EU.

### 2.2. Human Tau Oligomers and Tau Fibrils

Human tau oligomers and tau fibrils were obtained as a gift from Dr. Rakez Kayed (Galveston, TX, USA) and were prepared according to the previously published protocol [17,63]. In brief, recombinant human tau protein (tau-441 [2N4R], MW 45.9 kDa) was expressed and purified [64], and treated with 8 M urea to obtain monomeric human tau. Oligomers were generated by cross-seeding with Aβ oligomers. Aβ oligomers (0.3 mg/mL) were added as seeds, and the sample was mixed by pipetting and incubated for 1 h on an orbital shaker at RT. Purified human tau oligomers were used to seed a fresh monomeric tau sample. All Aβ oligomer seeds were eliminated after two rounds of seeding monomeric human tau with purified human tau oligomers. To prepare the fibrils, human tau oligomers were allowed to mix for 1–2 days on an orbital shaker.

### 2.3. Experimental Groups and Design

Animals (males, n = 90) were randomly assigned to three groups (sacrificed after 4, 8, and 11 months, respectively) and were unilaterally injected into the mEC with human tau oligomers, tau fibrils, or sterile saline solution (SSS). Each group consisted of 10 animals. Before each endpoint, animals were tested for the novel object recognition test (NORT), novel object location test (NOLT), and T-maze. To eliminate the possibility of detecting inoculated human tau, we also monitored tau changes 3 days after injection of human tau oligomers, human tau fibrils, and saline solution into the mEC (n = 18, each group consisting of 6 animals).

### 2.4. Stereotaxic Injections

Three- to four-month-old male Wistar rats were deeply anesthetized with isoflurane (2–3% in O_2_, 2 L/min) and placed in a stereotaxic apparatus (Kopf Instruments Model 940, Tujunga, CA, USA) with a heating pad to maintain body temperature. Anesthesia was maintained with 1% isoflurane in 0.5 L/min O_2_. Once the skull was exposed and cleaned, a burr hole was drilled over the appropriate coordinates and human tau oligomers, tau fibrils, or SSS were injected.

**Figure 1:**
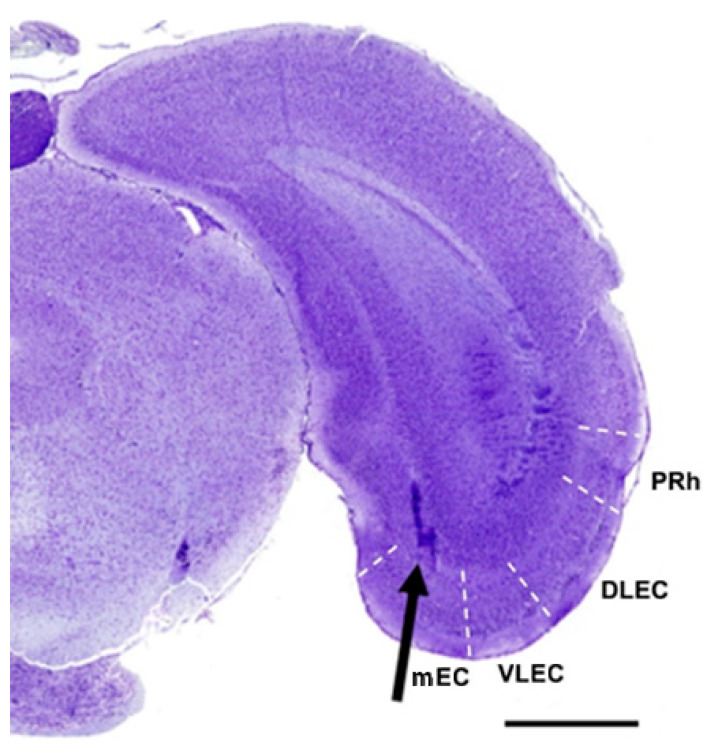
Schematic representation of stereotactic injection into the mEC in the right hemisphere of the Wistar rat and verification of position coordinates using Evans Blue. For the injection, the following coordinates were used: anterior-posterior −6.84 mm, medial-lateral 3.30 mm, dorsal-ventral 8.63 mm, angle 10°. The dashed lines are made according to the rat brain atlas, bregma −7.56 mm [65]. Nissl staining of rat brain after being sectioned showing the blue-colored tracer Evans Blue (black arrow). mEC, medial entorhinal cortex; DLEC, dorsolateral entorhinal cortex; PRh, perirhinal cortex; VLEC, ventrolateral entorhinal cortex. Scale bar = 2 mm.

Unilateral stereotaxic injections were performed in the mEC in the right hemisphere at the following coordinates: anterior-posterior −6.84 mm, medial-lateral 3.30 mm, dorsal-ventral 8.63 mm, angle 10°. Stereotaxic coordinates for the EC injection were obtained from a rat stereotaxic atlas [65] and injections at the correct coordinates were validated with the stereotaxic injection of Evans Blue (Figure 1).

Cortical connectivity of the mEC in rodents features interactions with areas such as the presubiculum, parasubiculum, retrosplenial and postrhinal cortex, all areas that are considered to belong to the “spatial processing domain” of the cortex [66]. In contrast, the lateral EC is strongly connected with olfactory areas, insular, medial- and orbitofrontal areas, and the perirhinal cortex—these areas are likely more involved in the processing of object information, attention, and motivation [66]. Therefore, from an anatomical and functional point of view, the procedure of injection of tau into the mEC mimics neurofibrillary changes that occur in early AD and is responsible for cognitive decline and spatial disorientation seen in humans [67].

A total of 4 µg of human tau oligomers in a total volume of 4 µL and a total of 4 µg of human tau fibrils in a total volume of 4 µL or SS (4 µL) were injected at a rate of 1 µL per min, using a 10 µL Hamilton glass syringe (Hamilton Company, Reno, NV, USA). After injection, the needle was left in place for 5 min before removal to prevent any leakage of the injected material. Rats were monitored during stereotaxic surgery by observing their breathing and monitoring toe reflexes. Rats were weighed and checked daily for 2 weeks following surgery, with access to food and water ad libitum.

### 2.5. Histology, Immunohistochemistry, and Staging

Rats were deeply anesthetized and transcardially perfused with ice-cold saline followed by 4% neutral buffered formalin, as previously described [68] at 4, 8, and 11 months post-injection. Brains were immediately removed and fixed overnight in 4% neutral buffered formalin before embedding in low-melting paraffin with a melting point from 52–54 °C (BioWax 52/54, BioGnost, Zagreb, Croatia) [69]. Paraffin blocks were serially sectioned using a microtome to produce 10-µm-thick tissue sections for conventional histochemical staining and immunohistochemistry. We used coronal sections to examine the eventual contralateral spread of the neurofibrillary and other changes.

To identify possible tau-related pathological changes, we used the Gallyas Braak silver staining method [70,71,72]. After sections were deparaffinized and immersed in distilled water (dH_2_O), they were placed in 5% periodic acid for 5 min and then washed twice in dH_2_O for 5 min. The sections were incubated in alkaline silver iodide solution (40 g sodium hydroxide, 30 g potassium iodide, 30.5 mL 1% silver nitrate, with dH_2_O, added to 500 mL) for 1 min and placed in 0.5% acetic acid for 10 min. The sections were developed in developer solution for 5–30 min mixed with 50 mL solution A (50 g sodium carbonate in 1000 mL dH_2_O), 15 mL solution B (2 g ammonium nitrate, 2 g silver nitrate, 10 g tungstosilicic acid in 1000 mL dH_2_O) and 35 mL solution C (2 g ammonium nitrate, 2 g silver nitrate, 10 g tungstosilicic acid and 7.3 mL 37% formaldehyde in 1000 mL dH_2_O). The sections were placed in 0.5% acetic acid for 3 min and then washed in dH_2_O for 5 min. The sections were incubated in 0.1% gold chloride for 5 min, rinsed briefly in dH_2_O, and placed in 1% sodium thiosulphate solution for 5 min. After washing in tap water, slides were dehydrated through a series of ethanol solutions (70% ethanol, 80% ethanol, 95% ethanol, and 100% ethanol) for 3 min each and cleared in 2 changes of xylene solution. Gallyas-stained coronal sections were selected at identical brain coordinates (anterior to the injection level, −5.64 mm from bregma; injection level, −6.84 mm from bregma; posterior to the injection level, −8.04 mm from bregma). Bregma levels of −8.04 mm, −6.84 mm, and −5.64 mm were analyzed for the spatial distribution of AT8-positive neurons [69].

We analyzed the progression of abnormally phosphorylated tau with the monoclonal antibodies (mAbs) AT8 (specific for tau phosphorylated at Ser202/Thr205/Ser208, 1:100, Thermo Scientific, Waltham, MA, USA, cat. no. MN1020, RRID:AD_223647) [73,74] and MC1 (specific for an NFT pathological conformation of tau, reactivity depends on both epitopes between amino acids 7–9 on N-terminus and 313–322 on C-terminus in the third microtubule domain, 1:20; a gift from Dr. Peter Davies, Feinstein Institute for Medical Research, Manhasset, NY, USA, RRID:AD_2314773) [75]. Antibody AT8 detects aggregated phosphorylated tau in the non-argyrophilic pre-tangle stage, whereas MC1 antibody reveals aberrant conformation of tau considered to be one of the earliest events in AD, which also correlates well with the severity and progression of AD [76]. It is present in a soluble form of the protein in paired helical filaments (PHFs) and precedes aggregation of tau into filaments [77]. To specifically detect human tau, we used the human tau-specific antibody HT7 (dilution 1:1000, human tau-specific antibody, aa 159–163, Thermo Scientific, Waltham, MA, USA, cat. no. MN1000, RRID:AD_2314654). To analyze whether inoculated tau proteins enter synapses and affect their decay, colocalization was performed with synaptophysin immunohistochemistry (SYN, 1:100, Dako, Glostrup, Denmark, cat. no. M7315, RRID:AD_2687942), a presynaptic protein that binds to presynaptic vesicles and with antibody T22 (1:200, Sigma-Aldrich, St. Louis, MO, USA, cat. no. ABN454, RRID:AD_2888681), which is specific for inoculated tau.

Tissue sections were deparaffinized in xylene and rehydrated through a series of increasingly diluted ethanol solutions. Slides were incubated in boiling citrate buffer (anhydrous citric acid solution 10 mM, pH 6; for AT8 0.05% Tween 20 was added) in a microwave at low power for 20 min. Endogenous peroxidase activity was inhibited by treating sections with 0.03% H_2_O_2_ in methanol for 30 min for MC1 and HT7; 0.07% hydrogen peroxide in dH_2_O for 15 min for AT8. Non-specific binding was blocked with 5% bovine serum albumin in phosphate-buffered saline (PBS) with Triton for MC1, 5% normal goat serum in PBS with Triton for HT7, and 10% normal goat serum in Tris-buffered saline for AT8 for 60 min. For SYN and T22, non-specific binding was blocked in 1% bovine serum albumin in PBS with 0.5% Triton for 120 min. After overnight incubation at 4 °C with primary antibodies diluted in blocking solution, slides were incubated with either horse anti-mouse or goat anti-rabbit antibodies (1:200) conjugated to biotin for 60 min (Vector Laboratories, Burlingame, CA, USA), followed by the application of the ABC complex (Vector Laboratories). The peroxidase activity was developed using 3,3′-diaminobenzidine as a chromogen and slides were mounted with Vectamount. As a negative control, sections were incubated in the absence of primary antibodies, and hippocampal slides from AD patients were used as positive controls. Every 20th coronal slide of the entire brain was stained and sections at −8.04, −7.32, −6.84, −6.00, and −5.64 mm from bregma were selected for further analysis. Thioflavin-S (ThS) staining was performed to visualize NFT and Aβ. For ThS staining, sections were incubated with 1% ThS in dH_2_O in the dark for 8 min. This step was followed by differentiation in two changes of 80% ethanol, two changes of 96% ethanol, and two washes in distilled water.

### 2.6. Behavioral Testing

Ten days before the onset of the behavioral testing, the rats were habituated to the testing room. One week before behavioral testing, rats were handled daily (15 min/day). Throughout the study period, animals were housed in a controlled environment (21 ± 2 °C, 50–60% relative humidity, 12 h light/12 h dark schedule). The maximum period in which behavioral tests were carried out was between 12 p.m. and 4 p.m. The order of the tests was as follows: T-maze test, NORT, and NOLT.

Rats were tested in the T-maze for rewarded alternation, as described previously [78,79]. The apparatus consisted of three identical arms (70 × 70 cm). The natural tendency of rats is to alternate their choice of goal arm [80] using spatial working memory [81]. If two trials are given in quick succession, on the next trial the rat tends to choose the arm not visited before, reflecting spontaneous alternation. In rewarded alternation test, the animal is rewarded for alternating. Animals were habituated on T-maze and for a taste of food reward for 5 consecutive days. Once the animals were habituated, ten trial sessions were performed per day, for 4 consecutive days. Animals started from the base of the T-maze and were allowed to choose one of the goal arms. An arm entry was scored when the animal placed all four paws within the arms. An alternation was defined as an entry to the previously non-visited arm in two given consecutive choices.

The NORT evaluates the recognition memory of one previously explored object compared with one novel object [82] and NOLT evaluates spatial memory where one object is being moved to a new location [83]. Rats were tested in an open-square white arena, 90 × 90 cm, and 70 cm high. Each animal was subject to habituation, exploration, novel object recognition, and novel object location. To minimize possible induced object preferences, selected objects were different enough so that rats could discriminate them easily. In this experiment, we used a rectangular box and a similar-sized oval container. Each trial was recorded and time spent exploring each object was measured manually by two independent observers (L.L.H. and E.Š.P.), as described earlier [83]. The task started with the habituation phase, during which the animals were placed in the empty arena for 10 min for 3 consecutive days. Twenty-four hours later, the rats were placed in the same arena containing two identical objects (familiarization phase) and exploration was recorded in a 5-min trial. Animals were considered to be exploring by sniffing, touching, and stretching their head toward the object. Distances shorter than 2 cm were scored as object investigation. Twenty-four hours later (test phase), rats were again placed in the arena containing one original object presented during the familiarization phase (familiar object), and a new object (novel object). Each animal was allowed to explore for 5 min and exploration of each object was manually recorded. During the novel object location, 24 h later, one of the original objects was displaced to a new location within the arena, and animals were allowed to explore for 5 min. The 24-h delay in retention testing was based on previous studies demonstrating object and location recognition [84]. For NOLT and NORT, the time exploring familiar and displaced/novel objects was expressed as a discrimination index, defined as the difference in exploration time for the displaced or novel object divided by total exploration time (seconds on novel/displaced − seconds on familiar)/(seconds on novel/displaced + seconds on familiar). This result can vary between +1 and −1. Animals with no memory impairment usually spend a long time investigating the novel/displaced object compared with the familiar object/location, giving a higher discrimination index. A negative score indicates more time spent with the familiar object/location, and a zero score indicates a null preference [85]. Increased time spent exploring the object in the novel location was interpreted as a successful spatial memory test, whereas increased time spent exploring the novel object was interpreted as successful recognition memory.

### 2.7. Statistical Methods

SPSS 19.0.1 (SPSS Inc., Chicago, IL, USA) was used for all statistical analyses. The level of statistical significance was set at α = 0.05. The results of animals’ behavior on cognitive tests between different groups were compared using ANOVA. Tukey’s multiple comparisons test was used for pairwise comparisons. If tests for equality of variances showed a statistically significant difference between variances of tested groups, a non-parametric Kruskal-Wallis test was used, and a post-hoc non-parametric test with a calculation of the corrected *p*-value for pairwise comparisons.

## 3. Results

### 3.1. Injected Human Tau Oligomers and Tau Fibrils Lead to the Appearance of Gallyas-Positive Inclusions in Wild-Type Rats

**Figure 2:**
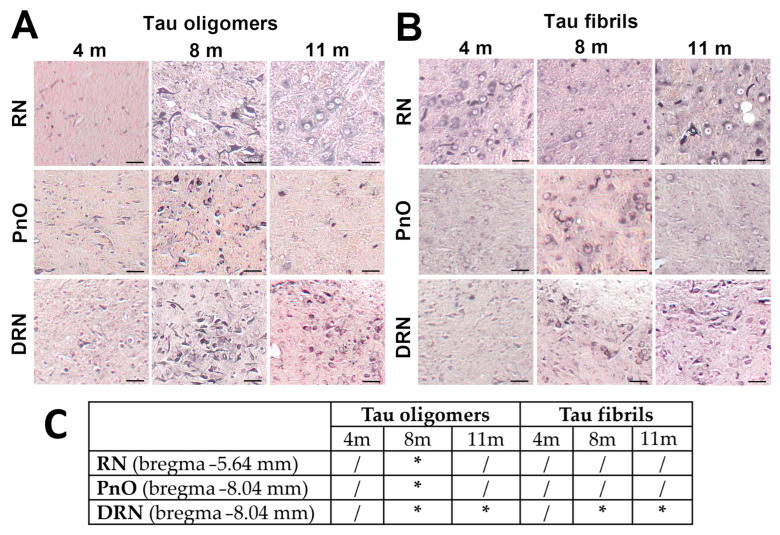
Tau protein-related changes after intracerebral injection of human tau oligomers (**A**) and tau fibrils (**B**) in the mEC on coronal brain sections of Wistar rats as revealed by Gallyas staining. (**A**) Gallyas-positive inclusions 8 months after injection of human tau oligomers were observed in the red nucleus and the PnO and the DRN 8 months and 11 months post-injection of human tau oligomers. (**B**) Gallyas-positive inclusions in DRN 8 months and 11 months after injection of human tau fibrils. (**C**) RN, red nucleus; PnO, pontine reticular nucleus; DRN, dorsal raphe nucleus. **/** denotes the absence of tau protein changes, * denotes the presence of tau protein changes. Scale bars = 50 µm.

We observed that delivery of human tau oligomers in the mEC induced the formation of Gallyas-positive inclusions in the red nucleus (RN) (at −5.64 mm from bregma) observable 8 months post-injection (Figure 2A), but not in the latter time-point (11 months). Neurofibrillary changes were not observed in the RN of animals injected with tau fibrils (Figure 2B). At the level of injection (at −6.84 mm from bregma), tau pathology was not observed in RN after injection of human tau oligomers and tau fibrils in any of the time points, probably because only a small quantity of human tau oligomers ended up in a few RN neurons at the selected level. We believe that no further pathological changes in tau proteins developed because these RN neurons are not susceptible to neurofibrillary degeneration.

Gallyas-positive inclusions were observed caudally to the injection site (−8.04 mm) in the pontine reticular nucleus (PnO) 8 months post-injection of human tau oligomers (Figure 2A). Comparable neurofibrillary changes in the dorsal raphe nucleus (DRN) were seen both at 8 and 11 months (Figure 2A) post-injection of human tau oligomers. Delivery of tau fibrils in the mEC likewise induced Gallyas-positive inclusions in DRN 8 and 11 months (Figure 2B) after injection. In animals who received tau fibrils, there was no tau-related pathology in the RN and PnO at any of the time points.

To confirm that the development of tau-related changes is not due to nonspecific effects of injection, rats were also injected with saline solution and Gallyas-silver staining was performed at the same levels and time points after injection. There were no tau-related changes observed in any of the control animals.

### 3.2. Injected Human Tau Oligomers and Human Tau Fibrils Initiate and Propagate Tau Pathology in Wild-Type Rats

**Figure 3:**
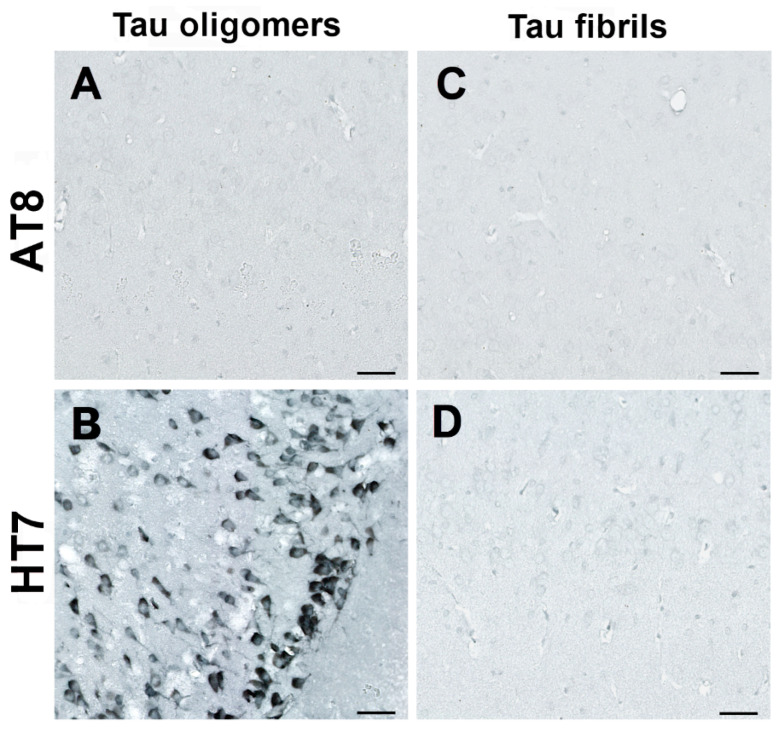
AT8 (**A**,**C**) and HT7 (**B**,**D**) immunoreactivity at the injection site (bregma −6.84 mm) 3 days after inoculation of human tau fibrils and tau oligomers into the mEC. (**A**) and (**C**) Three days after the injection of human tau oligomers (**A**) and human tau fibrils (**C**) there is no AT8 immunoreactivity around the injection site. (**B**) and (**D**) There is a lack of HT7 immunoreactivity 3 days after injection of tau fibrils (**D**), but strong immunoreactivity of the mEC neurons after the injection of human tau oligomers (**B**). Scale bars = 50 µm.

We monitored changes after injection using the human tau-specific HT7 and monoclonal antibody AT8 for phosphorylated tau to reveal the fate of inoculated human tau oligomers and fibrils. Three days after the injection of human tau oligomers no phosphorylated tau deposits were detected by antibody AT8 near the injection site (Figure 3A), but there was strong HT7 immunoreactivity in neurons of the mEC (Figure 3B). Three days after the injection of human tau fibrils, phosphorylated tau deposits were not detected by antibody AT8 around the injection site (Figure 3C) and there was also no HT7 immunoreactivity (Figure 3D). These findings suggest that inoculated human tau oligomers were taken up by local neurons of the mEC. As both human tau oligomers and human tau fibrils are non-phosphorylated they were not visualized by the AT8 antibody. Results obtained in Figure 3A,C also suggest that a 3-day time is insufficient for inoculated human tau oligomers and human tau fibrils to become phosphorylated at the AT8 epitopes or to induce phosphorylation of endogenous (murine) tau at the same epitopes.

**Figure 4:**
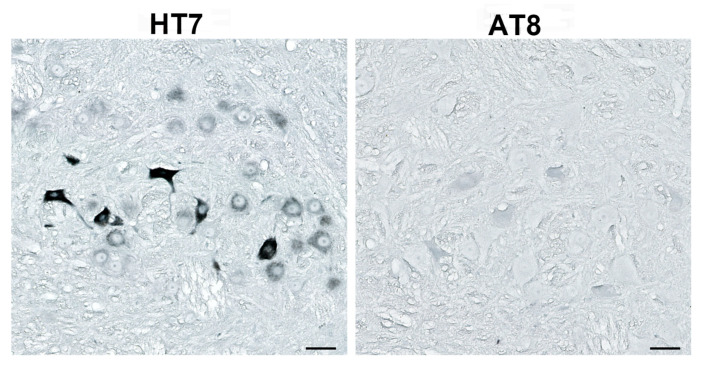
Three days after injection of tau fibrils (bregma −7.32 mm), tau seeds made from human aggregated recombinant tau (HT7 immunoreactivity) found in the PnO are not phosphorylated at the AT8 epitopes. PnO, pontine reticular nucleus. Scale bars = 50 µm.

We also compared HT7 immunoreactivity in the PnO to AT8 immunoreactivity (Figure 4) 3 days after injection of tau fibrils, which confirmed that tau seeds made from aggregated recombinant tau were not phosphorylated at AT8 epitopes. Altogether, these results suggest that AT8 immunoreactivity detected in later time points must represent phosphorylated endogenous rat tau recruited by the injected tau.

### 3.3. Propagation of Tau-Induced Changes

**Figure 5:**
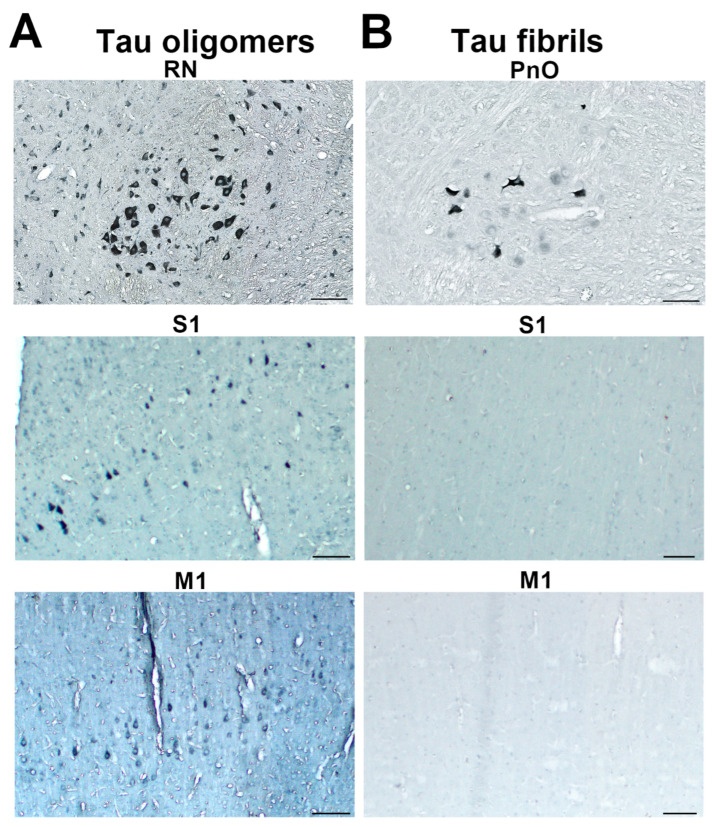
Propagation of human tau oligomers (**A**) and tau fibrils (**B**) 3 days post-injection in wild-type rats. (**A**) The primary motor and somatosensory cortex and the red nucleus are positive for human tau-specific monoclonal antibody HT7 3 days after injection of human tau oligomers (bregma −6.00 mm). Human tau oligomers are probably present in the red nucleus due to trans-synaptic transfer via the corticorubral tract, originating from the primary motor and somatosensory cortices. (**B**) Lack of HT7 immunoreactivity in the primary motor and primary somatosensory cortex is seen, but the PnO (at the level of bregma −7.32 mm) contains human tau-specific monoclonal antibody HT7 immunoreactive neurons. PnO, pontine reticular nucleus; RN, red nucleus; S1, the primary somatosensory cortex; M1, the primary motor cortex. Scale bars = 100 µm.

In animals injected with human tau oligomers, HT7 immunoreactivity was present 3 days post-injection in the primary motor and somatosensory cortices, as well as in the RN (Figure 5A). It is difficult to interpret this finding with absolute certainty. Human tau oligomers may have occurred in RN either due to the anterograde trans-synaptic spread of human tau oligomers from the primary motor and somatosensory cortices (these two regions receive projections from the mEC), but we cannot unequivocally reject that either anterograde trans-synaptic spread through other brain regions or by presynaptic fibers in the vicinity of the mEC that may have taken-up injected human tau oligomers then retrogradely transported to the RN. Three days after the injection of tau fibrils, we did not observe HT7 immunoreactivity in neocortical regions, but HT7 immunoreactivity was seen in PnO (Figure 5B).

### 3.4. Human Tau Oligomers and Tau Fibrils Induce the Formation of Conformationally Altered Murine Tau

**Figure 6:**
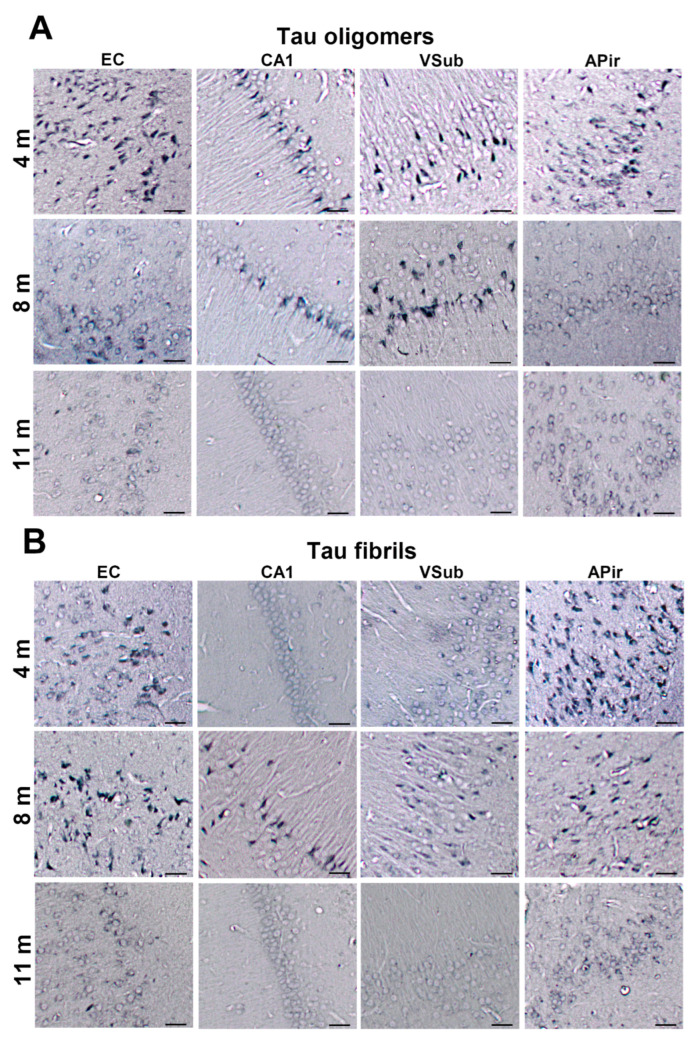
Conformationally altered murine tau (bregma −5.64 mm) after the intracerebral injection of human tau oligomers (**A**) and human tau fibrils (**B**), visualized with MC1 immunohistochemistry. EC, entorhinal cortex; CA1, CA1 field of the hippocampus; VSub, ventral subiculum; APir, amygdalopiriform transition cortex. Scale bars = 50 µm.

To determine whether a conformational change of the tau protein occurs after inoculation of human tau oligomers and tau fibrils, immunohistochemical analysis was performed using conformation-dependent anti-tau antibody MC1. Four months after human tau oligomer inoculation, MC1 immunoreactivity was observed throughout the EC, in the hippocampal CA1 field, ventral subiculum, and amygdalopiriform transition cortex (APir). After 8 months, immunoreactivity was still present in the EC, CA1 field, and ventral subiculum but was not observed in the Apir. After 11 months, weak MC1 immunoreactivity was present in the Apir, whereas it was not observed in other areas (Figure 6A). Four months after tau fibril inoculation, MC1 immunoreactivity was observed in the EC and Apir. After eight months, immunoreactivity was observed in the EC, CA1 field, ventral subiculum, and Apir. After 11 months, MC1 immunoreactivity was no longer present in any of these areas (Figure 6B).

### 3.5. Detection of Neurofibrillary Changes and Aβ with ThS

**Figure 7:**
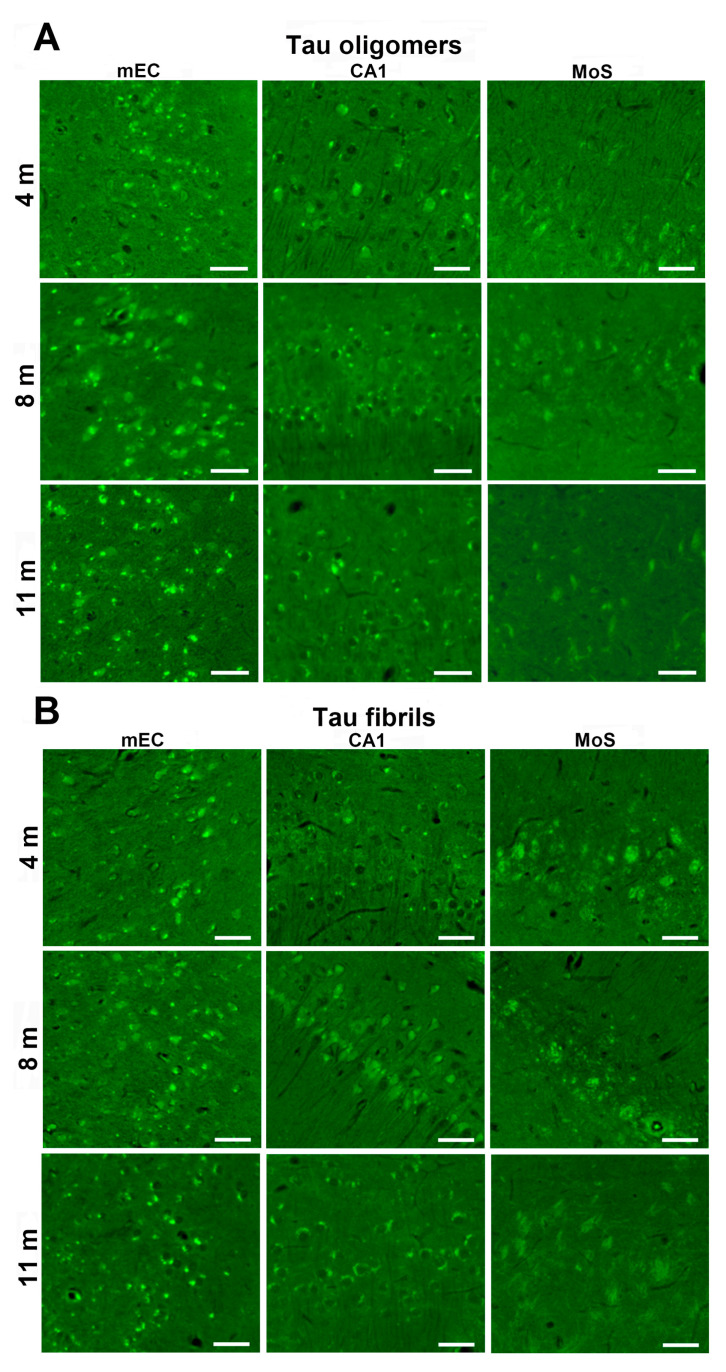
After inoculation of human tau oligomers, ThS-positive neurofibrillary changes and Aβ deposits are observed in the mEC, CA1 field of the hippocampus, and molecular layer of the subiculum of wild-type rats. (**A**) After inoculation of human tau oligomers, NFTs and Aβ were observed in the mEC and CA1 field of the hippocampus, whereas NFTs were observed in the molecular layer of the subiculum. (**B**) After inoculation of tau fibrils, mature NFTs and amyloid β were observed in the mEC and the CA1 field of the hippocampus, whereas ghost tangles and neuritic plaques were observed in the molecular layer of the subiculum. mEC, medial entorhinal cortex; CA1, CA1 field of the hippocampus; MoS, molecular layer of the subiculum; NFTs, neurofibrillary tangles; ThS, thioflavin-S. Scale bars = 50 µm.

After inoculation of human tau oligomers into the mEC, NFTs and Aβ were observed in the mEC and CA1 field of the hippocampus, whereas NFTs were observed in the molecular layer of the subiculum (Figure 7A).

After inoculation of tau fibrils, a signal corresponding to mature NFTs and amyloid β was observed in the mEC. Mature NFTs and amyloid β were observed in the CA1 field of the hippocampus, whereas ghost tangles and neuritic plaques were observed in the molecular layer of the subiculum (Figure 7B).

### 3.6. Synapse Loss in Rats Inoculated with Human Tau Oligomers and Human Tau Fibrils in the mEC

**Figure 8:**
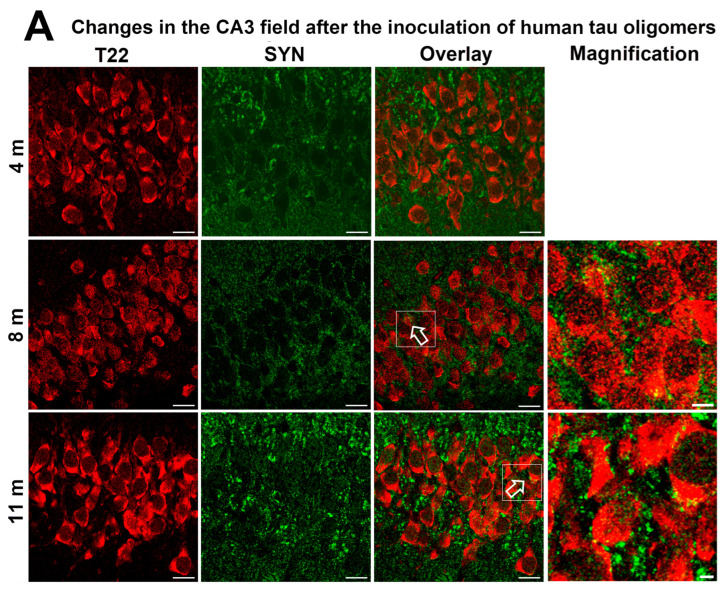

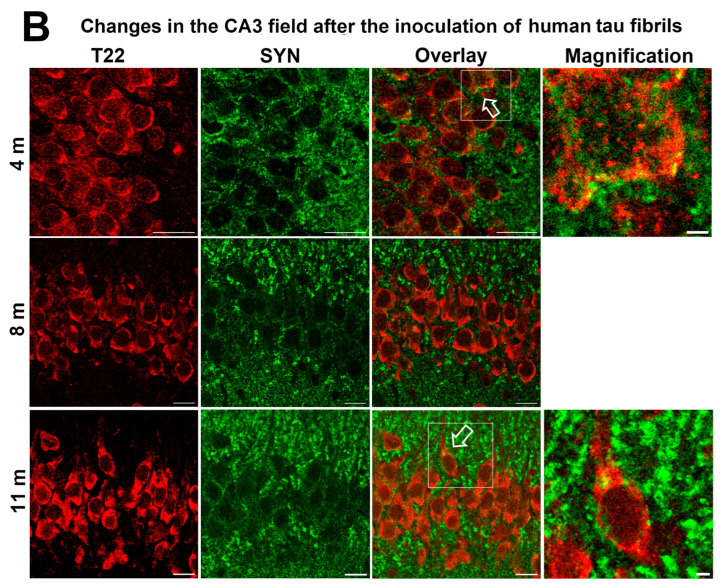
Colocalization of the presynaptic protein synaptophysin (SYN, green signal) with human tau oligomers and human tau fibrils (red signal) in the CA3 field of the hippocampus, as revealed by the T22 antibody. (**A**,**B**) Photomicrographs from the CA3 hippocampal field 4, 8, and 11 months after inoculation of human tau oligomers (**A**) and human tau fibrils (**B**). The open white arrow in the images shows the colocalization signal on the soma of the large pyramidal CA3 neurons (there are no colocalizations along dendrites). Magnified images in the last column show the appositions in the CA3 field of the hippocampus at 8 and 11 months (**A**, the last column) after inoculation of human tau oligomers. Magnified image of the colocalization signal in the CA3 field at 8 and 11 months (**B**, the last column) after the inoculation of human tau fibrils. Scale bars = 20 µm (the first three columns) and 2 µm (the last column).

Loss of synapses is an important pathogenic process influencing the onset of symptoms in AD [86]. We analyze whether human tau oligomers and human tau fibrils inoculated into the mEC enter hippocampal synapses and affect their decay using synaptophysin as a marker. Colocalization was observed in the hippocampal CA3 field 8 and 11 months after the inoculation of human tau oligomers into the mEC (Figure 8A) and 4 and 11 months after the inoculation of human tau fibrils into the mEC (Figure 8B), respectively.

### 3.7. Wild-Type Rats Injected with Human Tau Fibrils Display Rapid Propagation of Tau Protein-Related Changes Compared with Wild-Type Rats Injected with Human Tau Oligomers

**Figure 9:**
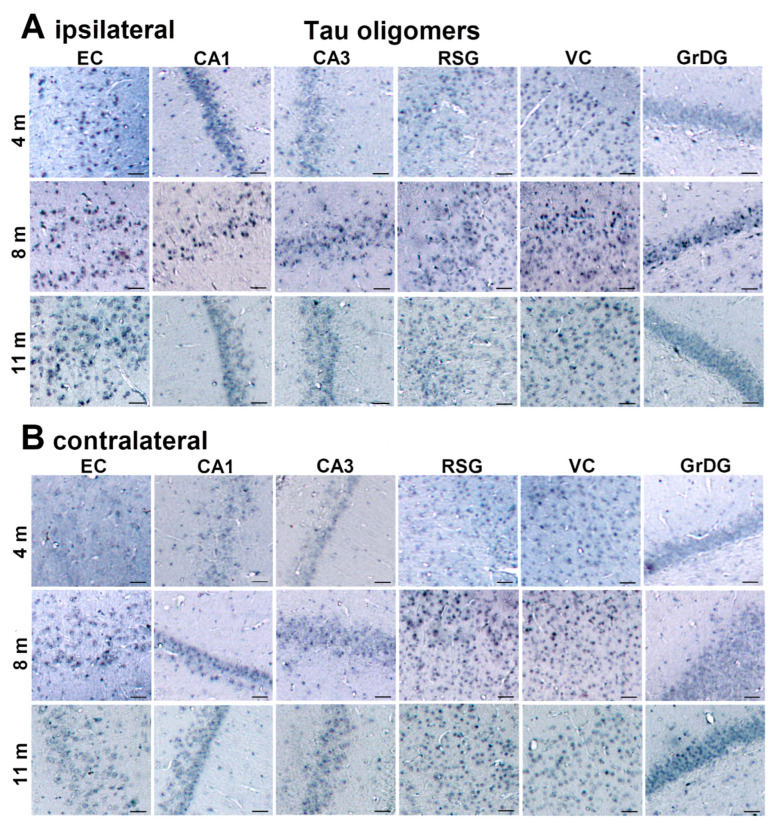

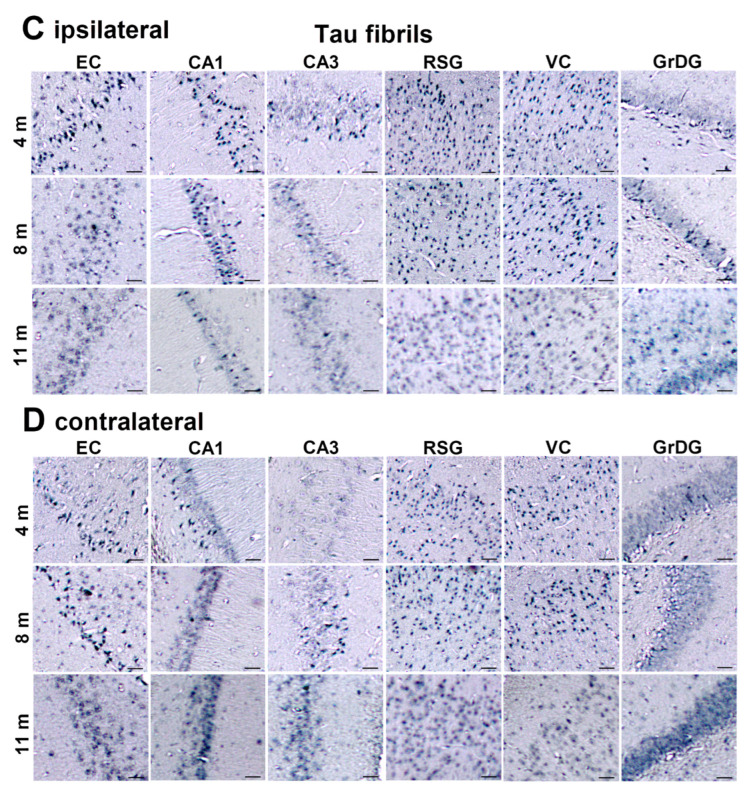
Progressive spread of neurofibrillary changes in the brain of wild-type rats identified by antibody AT8. AT8 immunostaining on the ipsilateral (**A**) and contralateral (**B**) side of the brain 4, 8, and 11 months after inoculation and tau fibrils on the ipsilateral (**C**) and contralateral (**D**) side 4, 8, and 11 months after inoculation. The representative images are given for the EC, CA1, and CA3 fields of the hippocampus, retrosplenial granular cortex, visual cortex, and the granular layer of the DG. CA1, CA1 field of the hippocampus; CA3, CA3 field of the hippocampus; DG, dentate gyrus; EC, entorhinal cortex; RSG, retrosplenial granular cortex; VC, visual cortex; GrDG, granular layer of the DG. Scale bars = 50 µm.

Using antibody AT8, serial coronal sections were analyzed to estimate the propagation of tau-related changes. Four months after inoculation of human tau oligomers into the wild-type Wistar rats, AT8 immunoreactivity was found in the EC, visual cortex, and CA1 field of the hippocampus. At eight months, AT8 immunoreactivity was observed in the EC, visual cortex, retrosplenial granular cortex, granular layer of the dentate gyrus (DG), and in the CA1 and CA3 fields of the hippocampus. Eleven months after inoculation of human tau oligomers, strong AT8 immunoreactivity was observed in the dorsolateral EC, whereas moderate immunoreactivity was observed in the visual cortex and retrosplenial granular cortex. Weak AT8 immunoreactivity was observed in the CA3 field of the hippocampus (Figure 9A).

Moreover, AT8 immunoreactivity spread to the contralateral side of the brain along cortico-cortical connections. At eight months, strong AT8 immunoreactivity was observed in the opposite hemisphere in the EC, retrosplenial granular cortex, and visual cortex, while weak AT8 immunoreactivity was observed in the CA1 and CA3 fields of the hippocampus. At 11 months, strong AT8 immunoreactivity was observed in the opposite hemisphere in the retrosplenial granular cortex, visual cortex, and granular layer of the DG (Figure 9B).

Four months after the administration of human tau fibrils, strong AT8 immunoreactivity was observed in the retrosplenial granular cortex, visual cortex, EC, granular layer of the DG, CA1 field, and CA3 field of the hippocampus. Eight months after the administration of tau fibrils, strong AT8 immunoreactivity was observed in the retrosplenial granular cortex, visual cortex, EC, granular layer of the DG, and CA1 field of the hippocampus, whereas weak AT8 immunoreactivity was observed in the CA3 field of the hippocampus. Eleven months after the administration of tau fibrils, AT8 immunoreactivity was observed in the retrosplenial granular cortex, visual cortex, and EC, whereas weak AT8 immunoreactivity was visible in the remaining areas assessed. A decrease in AT8 immunoreactivity was seen in all analyzed brain areas 11 months after fibrils were administered (Figure 9C).

As with human tau oligomers, the spread of AT8 immunoreactivity to the contralateral side of the brain was observed after the inoculation of human tau fibrils. Four months after human tau fibrils were inoculated, AT8 immunoreactivity was observed in the EC, retrosplenial granular and visual cortex, CA1 field, and the granular layer of the DG. After eight months, AT8 immunoreactivity spread to the CA3 field of the hippocampus. Eleven months after human tau fibrils were inoculated, AT8 immunoreactivity was still seen in all areas (Figure 9D).

### 3.8. Progressive Spread of Tau Protein-Related Changes to CA1 and CA3 Fields of the Hippocampus of Wild-Type Rats Are Identified by Gallyas-Braak Silver Impregnation

**Figure 10:**
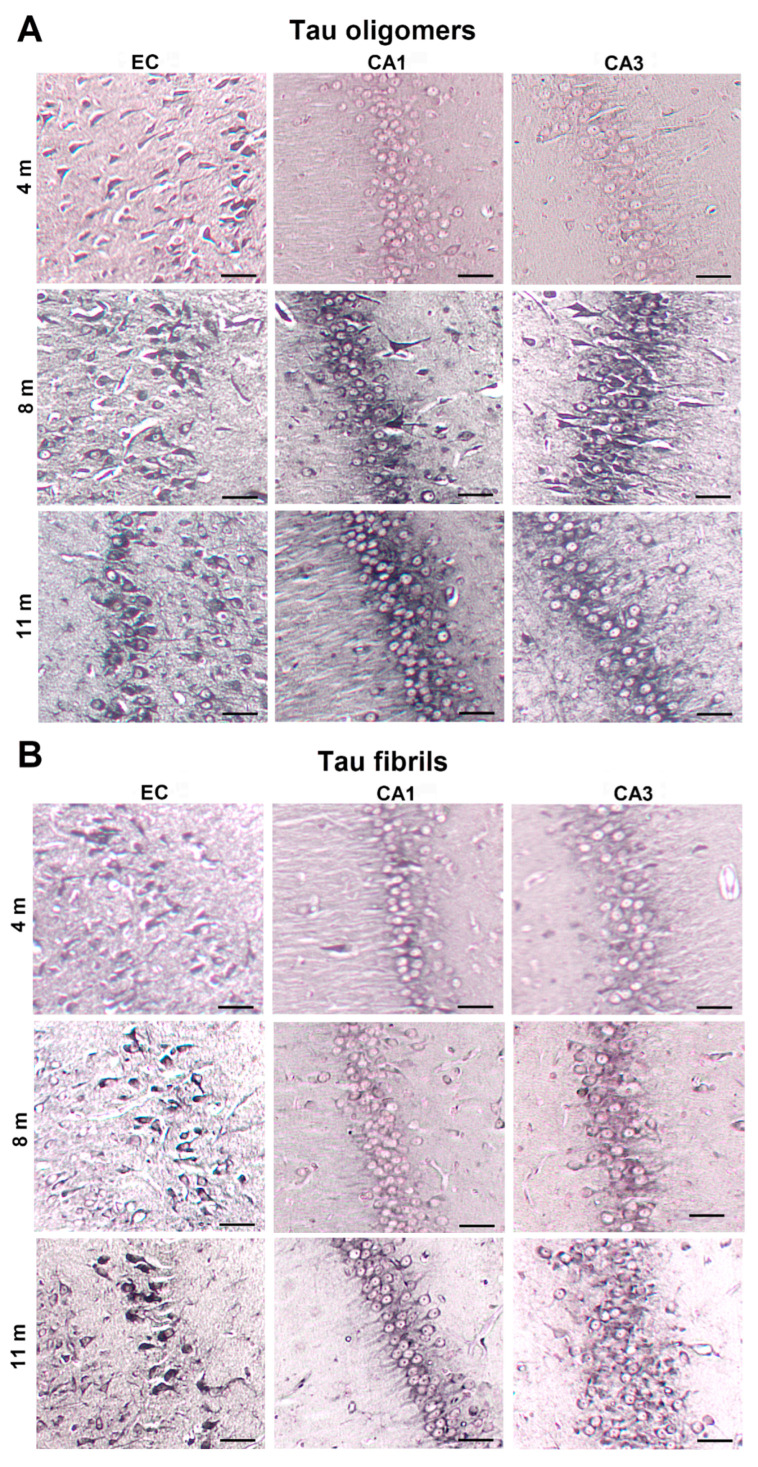
Progressive spread of neurofibrillary changes in the CA1 and CA3 fields of the hippocampus of wild-type rats identified by Gallyas-Braak silver staining. (**A**) Gallyas-Braak silver staining 4, 8, and 11 months after inoculation of human tau oligomers and (**B**) human tau fibrils observed in the EC, CA1 field, and CA3 field of hippocampus. Scale bars = 50 µm.

Four, 8, and 11 months after inoculation of human tau oligomers (Figure 10A) and human tau fibrils (Figure 10B) into the mEC, the formation of Gallyas-positive inclusions were observed in the dorsolateral EC. Inclusions were also observed in the CA3 and CA1 fields of the hippocampus 8 and 11 months after inoculation of human tau oligomers. Mature tangles were observed 8 months after inoculation of tau oligomers and argyrophilic NFTs were observed eleven months after inoculation in the CA3 field of the hippocampus (Figure 10A). After tau fibril inoculation, Gallyas-positive inclusions were observed in the CA1 and CA3 fields of the hippocampus at all time points (Figure 10B).

Based on phosphorylated tau (detected by antibody AT8) and Gallyas silver staining, Braak and colleagues identified five groups of neuronal tau pathology: (1.) pretangles (AT8 immunoreactivity); (2.) the onset of NFTs (AT8 immunoreactivity and weak Gallyas positivity); (3.) NFTs (AT8 immunoreactivity and Gallyas positivity); (4.) early ghost tangles (weak Gallyas positivity and AT8 immunoreactivity); (5.) extracellular ghost tangles (weak Gallyas positivity) [87,88]. Positive staining with Gallyas and AT8 antibody revealed the presence of phosphorylated tau spreading from the injection site to hippocampal CA3 and CA1 fields. The results showed that the majority of inclusions in the hippocampal region and mEC corresponded to the second Braak stage of tau pathology 4 months after inoculation of tau fibrils, and to the fourth Braak stage after 8 and 11 months. In contrast to human tau fibrils, after inoculation of human tau oligomers in the CA1 and CA3 fields of the hippocampus, tau pathology corresponding to stage 3 was not observed until 8 months, and stage 4 after 11 months.

### 3.9. Summary of Neurofibrillary Changes after Inoculation of Human Tau Oligomers

**Figure 11:**
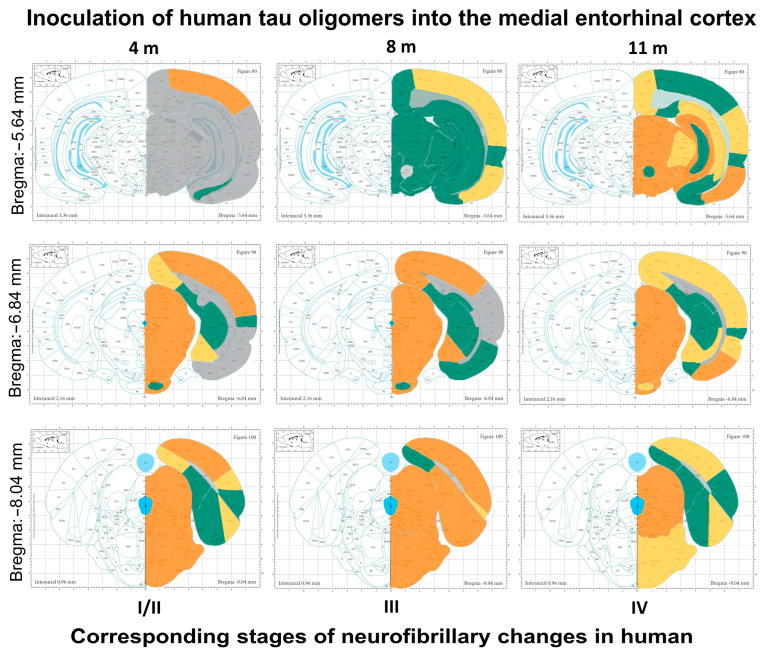
Propagation of neurofibrillary changes after inoculation of human tau oligomers using the AT8 antibody. The maps show the levels at the injection site (−6.84 mm from bregma), as well as one rostral (−5.64 mm from bregma) and one caudal (−8.04 mm from bregma) to the injection site. The semiquantitative analysis is represented by colors, as follows: grey—no AT8 immunoreactivity, green—weak AT8 immunoreactivity, yellow—moderate AT8 immunoreactivity, and orange—moderate to strong AT8 immunoreactivity. In the hippocampus and EC of rats inoculated with human tau oligomers from the fourth to the eleventh month, AT8 immunoreactivity weakened, and in parallel, the number of Gallyas-positive inclusions increased, a sequence of neurofibrillary changes comparable to the one seen in AD, where changes in stage I can be shown exclusively by AT8 immunohistochemistry. After the application of human tau oligomers into the mEC, the propagation of neurofibrillary changes in our model was slower compared to inoculation of tau fibrils (see Figure 12), as it took 11 months for neurofibrillary changes to spread across all areas of the rat brain. Corresponding stages of neurofibrillary changes in humans (stages I through VI) are labeled according to Hurtado et al. [69]. The left side of the figure contains the corresponding Paxinos and Watson atlas’ plates (with permission from “The rat brain in stereotaxic coordinates”, Sixth edition, Paxinos G. and Watson C., figures 80, 90, and 100, copyright Elsevier Academic Press, 2007).

In Figure 11, we summarized all changes through three time-points after inoculation of human tau oligomers into the mEC. Our results suggest that inoculation of human tau oligomers causes a progressive increase in phosphorylated and aggregated pathological tau and loss of synapses.

### 3.10. Summary of Neurofibrillary Changes after Inoculation of Human Tau Fibrils

**Figure 12:**
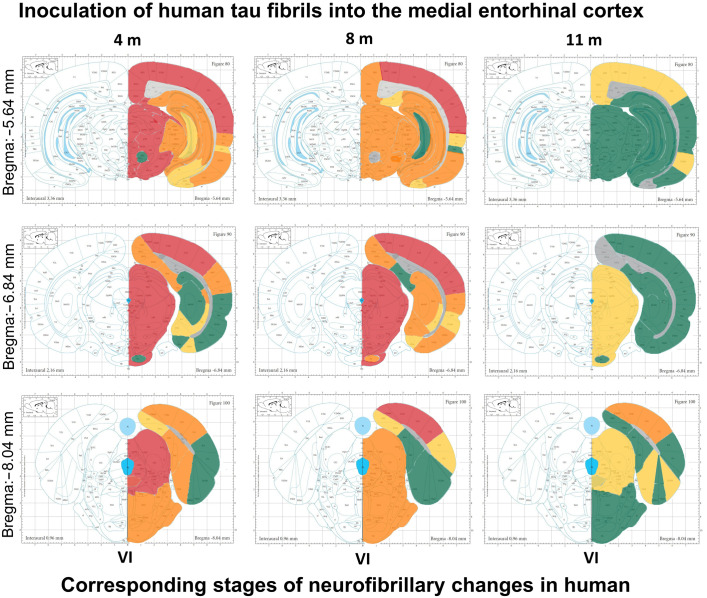
Propagation of neurofibrillary changes after inoculation of human tau fibrils using antibody AT8. The maps show the levels at the injection site (−6.84 mm from bregma), as well as one rostral (−5.64 mm from bregma) and one caudal (−8.04 mm from bregma) to the injection site. The semiquantitative analysis is represented by colors: grey—no AT8 immunoreactivity, green—weak AT8 immunoreactivity, yellow—moderate AT8 immunoreactivity, orange—moderate to strong AT8 immunoreactivity, and red—very strong AT8 immunoreactivity. Inoculated human tau fibrils induce and promote rapid propagation of neurofibrillary changes characteristic of AD that are observed very early in the hippocampus. Animals inoculated with tau fibrils show after 4 months a spread of phosphorylated tau protein at the AT8 epitopes in all brain areas, which corresponds to stage VI of human AD. The left side of the figure contains the corresponding Paxinos and Watson atlas’ plates (with permission from “The rat brain in stereotaxic coordinates”, Sixth edition, Paxinos G. and Watson C., figures 80, 90, and 100, copyright Elsevier Academic Press, 2007). Corresponding stages of neurofibrillary changes in humans (stages I through VI) are labeled according to Hurtado et al. [69].

In Figure 12, we summarized all changes through three time points after inoculation of human tau fibrils into the mEC. Inoculation of human tau fibrils rapidly induced dramatic changes as rats inoculated with human tau fibrils already after 4 months show the propagation of phosphorylated tau protein at the AT8 epitopes in all areas of the brain, corresponding to stage VI of human AD (Hurtado et al. [69]).

Although previously published studies did not unequivocally confirm the possibility of propagating tau fibrils between neurons, our results suggest that inoculated tau fibrils serve as a template for further induction and spread of identical changes in endogenous tau monomers in areas of the rat brain that are anatomically connected to the inoculation site in the mEC. This is consistent with previous in vivo research suggesting that tau fibrils may act as a seed for the propagation of neurofibrillary degeneration between neurons [14,42].

### 3.11. Behavioral Testing

**Figure 13:**
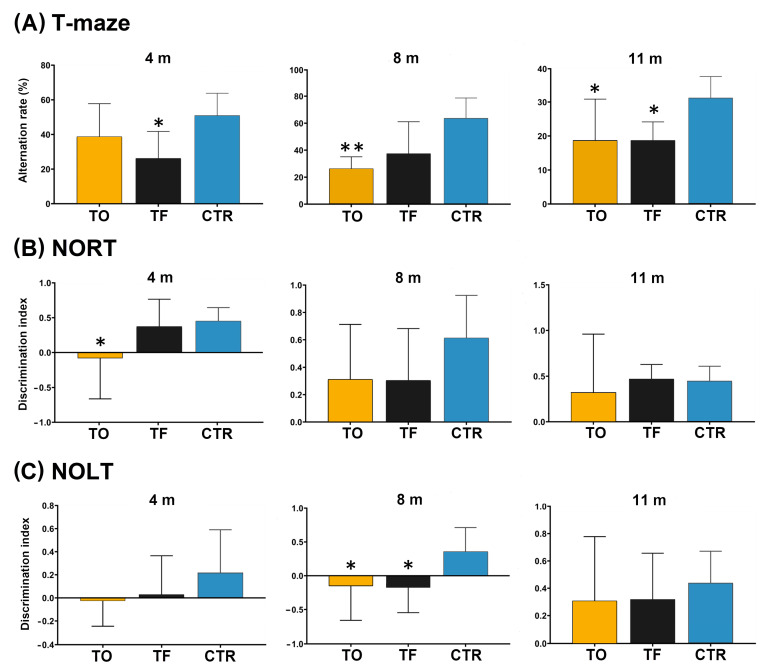
Behavioral testing of the animals that received human tau oligomers (TO), tau fibrils (TF), and controls that received physiological SSS (CTR). Testing included the T-maze rewarded alternation task (top panel), the novel object recognition test (NORT, middle panel), and the novel object location test (lower panel). The results are presented as the mean value ± SD. See text for details. CTR, control group; TF, tau fibril group; TO, tau oligomer group. * *p* < 0.05; ** *p* < 0.01 compared to CTR.

To evaluate different paradigms of memory and learning, a battery of three behavioral tests (T-maze, NORT, and NOLT) was completed 4, 8, and 11 months after intracerebral injection of human tau oligomers (TO group) and fibrils (TF group) into the EC, the major gateway between the hippocampus and the neocortex that, together with the hippocampus, has a critical role in memory, spatial navigation and multimodal integration (Figure 13).

The T-maze rewarded alternation task was used to assess the working memory of rats (Figure 13A). It revealed a significant difference in working memory 4 months after intracerebral injection of human tau fibrils and tau oligomers (F = 4.356, df = 2 22, *p* < 0.01). Tau fibril (*p* < 0.05) and tau oligomer (*p* < 0.05) injected animals displayed impaired performance in comparison to control animals (Figure 13A).

In the novel object recognition test (NORT), 4 months after injection of tau proteins, the rats from the tau oligomer group were unable to discriminate the novel object compared to the control group (F = 4.505, df = 2, 25, *p* < 0.05). The discrimination index showed significant differences after inoculation of human tau oligomers compared to the control group (Figure 13B). Long-term (24 h) object recognition memory was intact in both tau oligomer and tau fibril animals at 8 months (F = 2.533, df = 2, 20, *p* = 0.1; Figure 13B) and 11 months (F = 0.705, df = 2, 20, *p* = 0.51; Figure 13B) after the intracerebral injection.

The performance of rats in the novel object location test (NOLT) expressed as the Discrimination Index (DI) allows the discrimination between the novel and familiar location of the objects where a negative score indicates more time spent with the familiar object. DI revealed an increase in the investigation time of the familiar object 8 months after injection of human tau oligomers and tau fibrils (F = 4.816, df = 2, 26, *p* < 0.05). Control animals showed a clear preference for exploring the novel location of an object, whereas a negative score in DI indicated more time spent with the familiar object in rats injected with human tau oligomers (*p* < 0.05) and tau fibrils (*p* < 0.05) (Figure 13C). However, there was no significant difference in performance on the object location test in both human tau oligomer and tau fibril animals at 4 months (F = 1.469, df = 2, 24, *p* = 0.25; Figure 13C) and 11 months (F = 1.783, df = 2, 17, *p* = 0.2; Figure 13C) after the inoculation.

## 4. Discussion

The main result of our study is a comparable progression of neurofibrillary changes between the wild-type rat model of induced tauopathy and changes observed during AD progression. Interestingly, 3 days after the intracerebral injection of human tau oligomers and tau fibrils into the mEC, we detected the propagation of human tau oligomers along the mEC projections to other cortical regions, especially to the primary motor and somatosensory cortices. Furthermore, we also detected human tau oligomers in the red nucleus and human tau fibrils in the PnO 3 days after their inoculation into the mEC. As the EC is heavily connected to the primary motor and sensory cortical areas, as well as association areas [88], it is possible that through these projections human tau oligomers had been trans-synaptically transferred to the red nucleus via the corticorubral pathway. Because the primary motor and sensory cortices were devoid of inoculated human tau fibrils, it is more likely that tau fibrils were transferred from the mEC into the PnO via presynaptic brainstem fibers and active retrograde axonal transport, but not through corticoreticular projections.

The development of entorhinal to hippocampal neurofibrillary changes in both experimental conditions suggests the high vulnerability of the perforant path and hippocampus [89,90,91,92,93]. Therefore, our findings confirm that disruption of the perforant path is one of the key underlying pathophysiological events in the course of AD [94,95]. Furthermore, our results indicate that tau fibril inoculation promotes the rapid spread of early tau protein changes typical of AD, as demonstrated by the AT8 antibody, as these changes are observed very early in the hippocampus. The progressive spread of phosphorylated tau from the EC to the CA1 and CA3 fields of the hippocampus and the DG detected with the AT8 antibody 4 months after injection of tau fibrils correspond to the second Braak stage of human tau pathological changes. In addition, injection of human tau oligomers into the mEC induced AT8 immunoreactivity in the hippocampus after 8 months and corresponded to Braak stage 3 of tau pathological changes in humans. We also documented the spread of phosphorylated tau to the hippocampus of the opposite hemisphere. These results are consistent with the previously described sequence of changes in AD, according to which cells of layer III of the EC send projections to the contralateral hippocampal formation [96]. Phosphorylation of the AT8 epitope was also observed in the DG of the contralateral hemisphere, consistent with previous studies describing that the largest component of the EC projection is directed toward the DG [96,97,98].

It has been shown that human tau fibrils compared to tau oligomers cause increased accumulation of phosphorylated tau protein in the ipsilateral and contralateral cortex. The ipsilateral anteroposterior spread of endogenous tau aggregates in the cortex indicates the occurrence of neurofibrillary changes in associated projection neurons, whereas the contralateral spread indicates the involvement of neurons that represent the origin of commissural projections [99,100]. In a study by Vergara et al. [100], increased propagation of neurofibrillary changes in the cortex of the ipsilateral and contralateral hemispheres occurred after inoculation of PHFs isolated from AD brains into 5xFAD transgenic mice. These results suggest that the spread of pathological tau protein changes depends more on the connectivity of individual areas of the cerebral cortex than on the proximity of the inoculation site of pathological human tau oligomers or fibrils.

We also confirmed that tau fibrils act as a seed for the propagation of tau pathological changes in brain areas anatomically connected to the EC. This is in accordance with previous findings and suggests that tau fibrils can act as a seed to propagate neurofibrillary degeneration between neurons in vivo, although whether tau fibrils can be transported from one neuron to neuron has not been unequivocally confirmed [14,42,101]. Similar findings were obtained in a study on a transgenic mouse model of AD (PS19), where inoculating synthetic tau fibrils into the hippocampus stimulated changes in endogenous tau protein and their further spread to anatomically connected areas of the brain [43].

We also observed tau pathology using the Gallyas silver method, as well as by immunohistochemistry using AT8 and HT7 antibodies. Positive staining with Gallyas staining and AT8 for Ser202/Thr205/Ser208 phosphorylated tau established the presence of phosphorylated neurofibrillary changes and the spreading of the pathology to neighboring, connected regions to the injection site, as well as to contralateral distant nuclei downstream from the initial, first-order projection areas. These findings again indicate that the spread of tau pathology depends on connectivity and not proximity.

Three days after injection of human tau oligomers or tau fibrils, tau phosphorylated deposits were not detected by AT8 antibody around the injection site. Comparably to tau fibrils, tau oligomers, and normal endogenous tau are not phosphorylated at the AT8 site, so the later presence of AT8 immunoreactivity confirms that all tau pathology detected was specifically due to human tau oligomer/fibril templated recruitment of endogenous tau followed by spreading.

Stereotaxic inoculation of human tau fibrils and tau oligomers resulted in the appearance of conformationally altered tau protein (MC1) seen as NFTs. Interestingly, after inoculation of human tau oligomers, the MC1 epitope was observed in the hippocampus and EC before phosphorylation of tau protein at the AT8 epitope, consistent with data showing that human tau oligomers lead to the formation of an increased level of the aggregated form of tau protein and that the conformational change of tau protein precedes the formation of PHFs, which is one of the earliest pathological changes of tau protein in AD [77,101,102]. Another neuropathological feature of AD-like pathology is the presence of amyloid plaques. Accumulation of Aβ in the hippocampal formation and EC was observed by ThS staining 4, 8, and 11 months after tau fibril inoculation. In addition to the observed changes, neurofibrillary changes corresponding to the 2nd and 4th degrees in humans were found. After inoculation of human tau oligomers in the hippocampus and EC, amyloid plaques were observed after 8 and 11 months. At the same time, neurofibrillary changes were also observed in the hippocampus and EC, corresponding to the 4th-degree stage in humans. The correlation between the development of plaques and NFTs detected by ThS at 8 and 11 months indicates that Aβ can influence the formation of argyrophilic and ThS-positive neurofibrillary changes. Interestingly, amyloid plaques were detected with ThS in the hippocampal formation and EC 4 months after the administration of human tau oligomers, while neurofibrillary changes (identified by AT8 antibody) were not yet present at that time. These findings fit well into the modified amyloid cascade hypothesis, which assumes that over time, elevated levels of Aβ_1–42_ oligomers cause an inflammatory response and damage of neurons due to disturbances in neuron metabolism and ion homeostasis, and these changes simultaneously lead to altered activity of kinases that regulate tau protein phosphorylation and the emergence of neurofibrillary degeneration [103,104].

Ideally, animal models of AD should replicate not only the main neuropathological hallmarks but also reproduce at least some of the relevant cognitive impairments. We observed cognitive impairments and association with tau pathology after intracerebral injection of different tau species. Unlike seeded tau aggregates, injected human tau oligomers and human tau fibrils were not phosphorylated. Tau aggregates may grow by incorporating nonphosphorylated tau, which then undergoes a conformational change and becomes hyperphosphorylated. This phosphorylated tau can accumulate inside neurons and cause axon degeneration and synapse loss, causing disconnection and resulting in memory deficits and cognitive impairment. To determine whether inoculated human tau oligomers and human tau fibrils enter synapses and affect their decay, the presynaptic protein synaptophysin that binds to vesicles in presynapses and is associated with synaptic function was examined. Colocalization was observed in the hippocampal CA3 field 8 and 11 months after the inoculation of human tau oligomers and 4 and 11 months after the inoculation of human tau fibrils. The results obtained correlate with the resulting deficit in spatial working memory. Similar results were obtained by Lasagna-Reeves and colleagues, who observed the influence of human tau oligomers on the reduction of presynaptic density and the cognitive deficit measured in NORT, whereas the influence of human tau fibrils was not observed [18].

In our experiments, rats injected with human tau oligomers showed impairments in spatial working memory in the T-maze rewarded alternation task 8 and 11 months post-injection, whereas 4 months after injection of human tau oligomers spatial working memory was not affected. These results are consistent with the lack of AT8 immunoreactivity in the hippocampus 4 months after inoculation of human tau oligomers, and severe Gallyas positivity and AT8 immunoreactivity in the hippocampus 8 and 11 months after inoculation. Cognitive impairment 4 months post-injection of human tau fibrils is consistent with AT8 immunoreactivity and Gallyas staining seen in the hippocampus. Eleven months post-injection, animals with cognitive impairment showed Gallyas-positive argyrophilic neurofibrillary changes and AT8 immunoreactivity in the hippocampus. We concluded that the correlation of tau phosphorylation recognized by the AT8 antibody in the hippocampus and impairment in the T-maze is comparable to previous studies showing that a deficit in spatial working memory is directly correlated to hippocampal dysfunction [105,106]. In general, these findings suggest that the accumulation of neurofibrillary pathology correlates with the severity of cognitive impairments and is in broad agreement with previous findings [34]. The NOLT showed impairments in spatial memory 8 months after inoculation of human tau oligomers and fibrils, a finding consistent with the tau pathology seen in the CA1 and CA3 fields of the hippocampus. This result is also in agreement with previous reports that the hippocampus is required for encoding, consolidation, and retrieval of novel object location, and is sensitive to manipulations of the dorsal CA1 field [107,108]. The novel object recognition test showed that the alteration of long-term memory occurred only 4 months after the inoculation of human tau oligomers. In parallel with the cognitive deficit, inoculation of human tau oligomers leads to the appearance of AT8-immunoreactive changes in the perirhinal cortex, which is consistent with a study performed in mice, in which it was shown that the perirhinal cortex is important for the correct perception of the object, i.e., receiving the necessary visual, olfactory, and somatosensory stimuli and their transmittance to the hippocampus [109]. In most tauopathies, wild-type tau aggregates into filaments, but neurofibrillary degeneration associated with wild-type tau protein is poorly represented by the currently available models that express mutated tau proteins in transgenic animals [11,110]. A major limitation of these models is that they exhibit pre-tangle stages without the full development of neurofibrillary degeneration and cognitive deficits comparable to those observed in humans [111,112,113,114,115].

### 4.1. Limitations of the Study

The major limitation of our study is that female rats were not included. Because the model was a first-time implementation, we wanted to minimize confounding outcomes due to hormone fluctuations during the female reproductive cycle. In future studies, female rats will be included and analyzed alongside male rats to address the potential roles and benefits of hormones in this model of tau propagation. This model could also contribute to the determination of new potential therapeutic targets for the suppression or prevention of the propagation of pathological tau protein changes. Limiting the spread of the abnormal and hyperphosphorylated tau proteins could have major therapeutic potential for AD and other primary and secondary tauopathies. One possibility of doing so is through immune therapy with tau antibodies. It was shown that after the injection of oligomers into the brain of hTau mice, antibodies against oligomeric tau (TOMA) can reduce the seeding of tau [116]. Furthermore, it has been shown that passive strategies using the PHF1 antibody and conformation-dependent anti-tau antibody MC1 can enhance rodents’ cognition and behavior [117]. Therefore, future studies should also test some of the before-described antibodies against tau isoforms in this model. This model could also assess different pathways and mechanisms of tau spreading that were not yet investigated, including mechanisms of tau secretion and tau uptake.

Another possible limitation of this study is the specificity of antibody AT8. Although antibody AT8 recognizes tau protein phosphorylated at Ser202 and Thr205, it does so also when tau is phosphorylated at Ser208 residue. Thus, it is still not fully resolved whether the phosphorylation of Ser208 residue is responsible for the pathological character of early tau protein change [118] and whether is it a reversible change in human individuals under the age of thirty [119]. In this respect, other stainings that reflect pathological changes of tau proteins, such as the Gallyas stain, are very helpful. However, although the injection of human PHFs made of tau proteins leads to the appearance of Gallyas-positive neuronal and glial inclusions in rodents [120], it is still not known whether Gallyas-positive grains are composed of solely conformationally changed and hyperphosphorylated murine tau proteins or also contain human tau proteins. This is an important issue to address in the context of differences in tau conformations that are relevant for interference in cross-seeding between human and murine tau proteins.

### 4.2. Future Perspectives

Despite these limitations, our study highlights several reasons for the development of new models of sporadic tauopathies in wild-type non-transgenic rats for the investigation of specific mechanisms regulating tau pathology. First, most transgenic animals fail to model disease progression from the pre-tangle stage to ghost tangles [121]. Second, rat as a model for tauopathies has some major advantages over other species. Rats are physiologically, and genetically less distant from humans than mice and they express six isoforms of tau proteins [7,122]. Rats have more complex central nervous systems, with longer postnatal brain development, and they show richer behavioral displays and higher cognitive abilities than mice [123,124]. Altogether, rat as a model of AD allows more detailed behavioral analysis and will thus enable a more accurate assessment of the impact of tau pathology on cognitive decline in future studies.

## 5. Conclusions

Inoculation of tau human tau oligomers and tau fibrils caused the progression of neurofibrillary changes from the mEC to other connected brain regions, similar to AD-related changes outlined in the human brain. The development and spreading of the earliest tau pathological changes, as revealed by using the AT8 antibody, occurred much faster in animals inoculated with human tau fibrils than in animals inoculated with human tau oligomers. The absence of tau pathology in the primary motor and sensory cortices upon human tau fibrils injection could be explained by the evidence that shows exogenously provided small, soluble tau oligomers but not tau fibrils, are much more efficiently internalized by neurons [125]. In addition to phosphorylation at the AT8 epitopes, stereotaxic inoculation of human tau fibrils and tau oligomers caused the appearance of conformationally altered tau protein, as revealed by using the MC1 antibody, and the formation of Gallyas-positive inclusions that confirmed the accumulation of pathologically changed tau proteins, as well as accumulation of amyloid β, loss of synapses in the hippocampus, and significant corresponding cognitive deficits documented by the NOLT, NORT, and T-maze test.

## Data Availability

The data presented in this study are available on request from the corresponding author.

## List of Abbreviations

Aβ	amyloid β
AD	Alzheimer’s disease
APir	amygdalopiriform transition cortex
APP	amyloid precursor protein
AT8	antibody specific for tau phosphorylated at serine 202, threonine 205, and serine 208
CA	*Cornu Ammonis* (Ammon’s horn)
CTR	control group
DG	dentate gyrus
DI	discrimination index
DLEC	dorsolateral entorhinal cortex
DRN	dorsal raphe nucleus
EC	entorhinal cortex
FTDP-17	frontotemporal dementia with parkinsonism linked to chromosome 17
GrDG	granular layer of the dentate gyrus
HT7	human tau-specific antibody
M1	primary motor cortex
MAPT	microtubule-associated protein tau
MC1	antibody specific for a pathological conformation of tau, binds to epitopes between amino acids 7–9 and 313–322
mEC	medial entorhinal cortex
MoS	molecular layer of the subiculum
MRI	magnetic resonance imaging
NIA-AA	National Institute on Aging and Alzheimer’s Association
NFT	neurofibrillary tangles
NOLT	novel object location test
NORT	novel object recognition test
PBS	phosphate-buffered saline
PET	positron emission tomography
PHF	paired helical filaments
PnO	pontine reticular nucleus, oral part (*nucleus reticularis pontis oralis*)
PRh	perirhinal cortex
RN	red nucleus
RSG	retrosplenial granular cortex
S1	primary somatosensory cortex
SSS	sterile saline solution
SYN	synaptophysin T22 antibody that specifically recognizes oligomeric tau
TF	group of animals that received tau fibrils
ThS	thioflavin-S
TO	group of animals that received tau oligomers
VC	visual cortex
VLEC	ventrolateral entorhinal cortex
VSub	ventral subiculum
